# The effects of ship wakes in the Venice Lagoon and implications for the sustainability of shipping in coastal waters

**DOI:** 10.1038/s41598-019-55238-z

**Published:** 2019-12-12

**Authors:** Gian Marco Scarpa, Luca Zaggia, Giorgia Manfè, Giuliano Lorenzetti, Kevin Parnell, Tarmo Soomere, John Rapaglia, Emanuela Molinaroli

**Affiliations:** 1 Università Ca’ Foscari, Dipartimento di Scienze Ambientali, Informatica e Statistica, Venezia, 30175 Italy; 2 Consiglio Nazionale delle Ricerche, Istituto di Scienze Marine, Venezia, 30122 Italy; 3 Tallinn University of Technology, Department of Cybernetics, Tallinn, Estonia; 4 Sacred Heart University, Department of Biology, Fairfield, CT 06825 United States of America

**Keywords:** Environmental impact, Hydrology, Physical oceanography

## Abstract

We analyse the impact of ship traffic in the vicinity of navigation channels in a wide shallow waterbody. The crucial hydrodynamic driver in this situation is the depression (Bernoulli) wake that may be transferred into a long-living solitary wave of depression over the shoals. The analysis considers navigation channels in the Venice Lagoon using a new large dataset of approximately 600 measured wake events associated to specific ships whose data are provided by the AIS system. Since the development of the modern industrial port and the opening of the Malamocco–Marghera channel in the late 1960s, growing pressure on the lagoon caused by ship traffic has raised concerns about its physical integrity and habitat survival. The transit of large vessels has been shown to have serious impacts on the shallow water areas adjacent to waterways. Depression wakes created by such vessels can reach significant dimensions (water level dropdown of up to 2.45 m at the channel margin), causing unusually large retreat rates of several sections of the shoreline and which may adversely affect the lagoon morphology. The wakes are analysed in relation to ship and morphological parameters. A formulation is proposed to predict wake amplitude on the basis of ship characteristics and motion.

## Introduction

Venice, was famously called the “Queen of the Seas”, thanks to its history as a maritime superpower^1^. For centuries, starting from the late medieval period, its port was the focus of the “global” economy”^2^. Venice’s growth and power as a prosperous trading place and maritime empire in the eastern Mediterranean, was due to the accessibility of its docks, which was made possible by a continuous monitoring of the environment and direct interventions to control the morphological evolution of its lagoon system consisting of inlets, barrier islands and tidal channels^3,4^.

**Figure 1:**
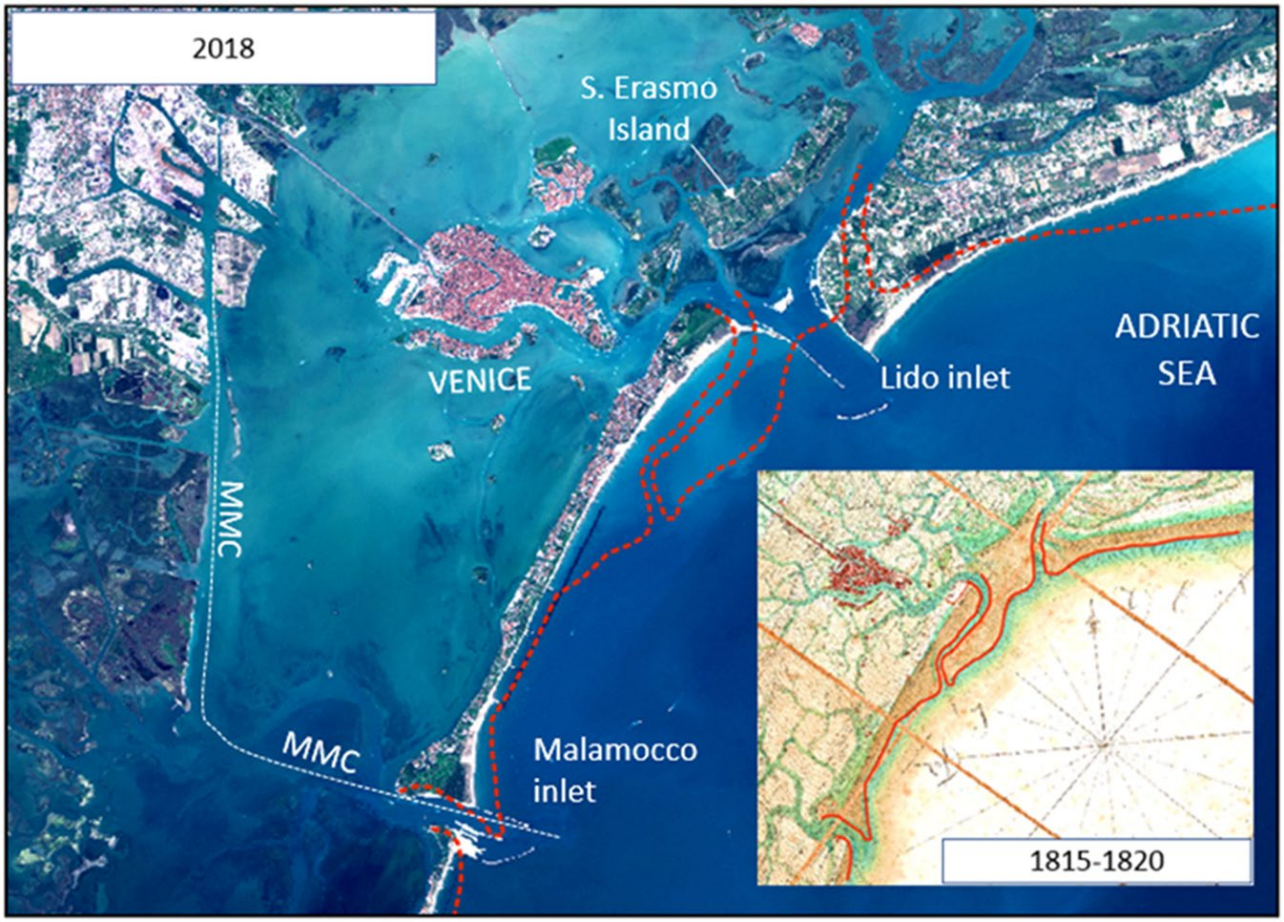
Red dotted lines represent the shoreline position in 1815–1820 taken from the Map of the Lagoon by Combatti Bernardo (inset). The white line represents the path of the MMC channel. The large white structures at west of the Venice centre are the docking piers of Stazione Marittima, the current passenger terminal. The pseudo-true-colour Copernicus Sentinel-2b image (S2B_MSIL1C_20180414T101019) was acquired on 14 April 2018, downloaded from https://eros.usgs.gov/about-us/data-citation under EU open access policy (https://sentinel.esa.int/web/sentinel/sentinel-data-access). The satellite image was processed using Copernicus Sentinel 2018 data (L1C level of processing).

For generations, the morphology of the lagoon and its inlets dictated ship characteristics and provided the basis for the city’s defence strategy. Navigation and coastal engineering therefore adapted to the natural trends in morphology and hydrodynamics. The map of the lagoon^4^, made by Bernardo Combatti in 1815–1820 (inset in Fig. 1), clearly shows the presence of a sand spit at the Lido inlet and signals the intense sand transport along the coast to the southwest.

A radical solution was implemented in 1897, when three inlet channels close to the city were joined to form a single large inlet with long jetties that extended over the pre-existing ebb-tidal delta and incorporated the barrier island of S. Erasmo into the lagoon^5^. The increased tidal flow through the new inlet channel of Lido successfully prevented its further silting and the channel only required limited dredging. However, the jetties also blocked the longshore sand transport, leading to aggradation on the updrift beaches and severe erosion downdrift of the structures^6^.

The stabilization of the port channel gave Venice the impetus for a new phase of development as an industrial port from the early twentieth century. Ships could access the port terminal of “Stazione Marittima” (located just west of the city centre, Fig. 1), and from 1922 the canal was extended from Stazione Marittima to the Porto Marghera Industrial Zone (PMIZ) which was built in that period at the mainland-lagoon interface^7^. For four decades, the route to the PMIZ passed through the historic centre of Venice. Pressure on the city was reduced, however, by the construction of a new waterway, the Malamocco–Marghera Channel (MMC, from the inlet of Malamocco to the PMIZ industrial district), through the central lagoon, in the late 1960s.

After the opening of the MMC, industries in Marghera expanded rapidly and Venice became the principal port of the northern Adriatic with a current number of about 3500 port calls. Among these ~3000 (essentially all commercial vessels) sail through the Malamocco inlet^8^ and ~500 (mostly cruise ships) through the Lido inlet. Ship size has progressively increased as has the volume of traffic leading to serious concerns regarding the impacts of shipping, such as pollution and erosion induced by ship wakes, as well as the overall need to protect the lagoon for the safeguard of the historic city of Venice (see as references: https://weareherevenice.org/wpcontent/uploads/2017/05/IMG_6951.jpg; https://www.theguardian.com/travel/2017/may/26/venice-tourists-cruise-ships-pollution-italy-biennale). These impacts are generic and present in similar locations worldwide.

Vessel wakes generated in open sea areas decay rapidly with distance from the ship^9,10^ and have negligible impact on the seabed, on the nearshore area, and on ecosystems. In shallow coastal areas and narrow waterways, however, the wake generation mechanisms, propagation patterns and impact modes may radically deviate from those typical for open sea conditions. It is well known that ship traffic can significantly damage vulnerable areas such as tidal creeks^11^, microtidal estuaries^12^, lakes^13,14^ or wetlands^15,16^. The impact of vessel wakes can be the most significant factor for changes near fairways, such as on the channel edges in salt marshes^17^, on the shores of navigable delta channels^18^, and on river banks^19^.

Ships sailing at even moderate speeds in shallow areas or channels may excite specific types of disturbances such as dangerously high leading (solitary) waves^20^, monochromatic packets of very steep and short waves^21^, depression areas^20^, or supercritical bores^22^. This not only jeopardizes the safety of smaller vessels in the vicinity but may also seriously damage the coastal environment^23,24^ and impact the integrity of the bottom^12^. Depressions originating in the channels of the Venice Lagoon may transform into strongly nonlinear deep solitary waves of depression^25,26^ that propagate over a long distance across the lagoon^27^ and resuspend large amounts of sediment along their way^28,29^. In addition, the human-induced components of hydrodynamic activity and associated enhanced erosion may affect not only the integrity of the sea bottom but also (pre)historic cultural layers^30^. As these disturbances are qualitatively different from the usual wind waves or constituents of the linear Kelvin wake^23^, their evolution, interactions and impacts are thus different from the behaviour of linear waves.

The effects of navigation in restricted waterways have been widely investigated and the associated threats are well understood in navigation channels leading to ports^31^, rivers^32–35^ and narrow straits^36^ that are often used as major routes for commercial traffic. A specific feature of navigation channels in the Venice Lagoon is the presence of expansive tidal flats on one or both sides of the channel banks along extensive sections of the waterway. This gives rise to far-reaching effects of ship traffic that extend to a distance of a few kilometres from the fairway. Even though similar situations occur in several other geographical areas^37,38^, the specific features and threats of the hydrodynamic regime and associated sediment dynamics in a system that includes a channel and adjacent shallow water areas subjected to ship-induced depression wakes were identified only recently^25^.

As a consequence of the opening of the MMC and the growth in port traffic, the morphology of the Venice lagoon has significantly changed. Particularly, the central lagoon basin was affected by extensive erosion from 1970 to 2000^39^. The distribution of depths in this sub-basin underwent a significant change with the primary depth frequency moving from −0.80 m to −1.80 m^40^. About 80% of its surface suffered high or moderate erosion rates making it the most unstable area of the lagoon. The study of sedimentological processes also shows that the characteristics of this area are more similar to those of an open bay than of a lagoon environment^41^. Previous studies attributed the observed erosion to changes in the overall hydrodynamic regime of the lagoon induced by the new artificial channel^42^. Recent research indicates, however, that some of the erosion was caused by ship traffic^43^. In the light of the above discussion, it is not unexpected that the bottom, shores, edges of channels and the entire ecosystem of the Venice Lagoon may suffer from a number of the above-listed impacts associated with wave propagation and erosion on the channel sides and over the mudflats. The most significant causal factors are strongly nonlinear local depressions created by vessels in transit^28^. These can reach amplitudes of 2.5 m in the MMC channel^26^ and after shoaling on the channel side banks, propagate over a large distance on the sub-tidal flats, causing significant sediment resuspension^29^ from the shallow parts of the lagoon and fast erosion of channel sides and shorelines^43^.

Traffic in the industrial channel is expected to increase in the future, including plans to increase the maximum ship size. These developments further intensify the widespread concern about cruise ships transiting through the historic centre of Venice and the traffic in the MMC. Venice faces a difficult choice between its role as a port city and protection of the lagoon and its unique cultural heritage as the large scale and intensity of port traffic combined with the effects of navigation through a shallow lagoon are a severe threat to the integrity of the lagoon ecosystem^44^. The destiny of the city of Venice has always depended on the functioning of this ecosystem, that is already challenged and susceptible to global changes^44,45^. Understanding and managing the interactions between ship traffic and the environment is thus a priority for the sustainable management of the pressures of port activities in this location, as well as in other parts of the world.

The aim of this study is to integrate new measurements with the previous ten years of research on ship wakes and their impacts on the environment, and to identify critical factors affecting the geomorphology of the Venice lagoon. The focus is on the re-interpretation of several previously fragmented data sets, and to improve our understanding of the recent evolution of the channel-tidal flat system in locations where change is driven by the effects of ship traffic through the mechanism of highly nonlinear wave fields, specifically, long-living depression waves^25–27^. To achieve this, real-time ship traffic data from the Automatic Identification System (AIS) were integrated with instrument measurements. A cluster of sensors deployed in the area measured water level, current speed, turbidity and suspended sediment concentration. The resulting dataset contains information about the properties and impact of approximately 600 depression events. The recorded events are linked with the ship information. This made it possible to evaluate, for the first time, the relative importance of each variable (ship position, dimensions and velocity). This information can be used to predict the effects of increased traffic through the MMC, especially with a re-routing of cruise ships.

## Methods

Position and speed data that are transmitted, on average, once every 60 s from most commercial and passenger vessels, were acquired by a dedicated receiver located in the Acqua Alta oceanographic tower off the Gulf of Venice, transferred to a server and analysed with the Software AIS-Decoder. The resulting database contains the relevant information for ships located between 12.0° and 12.7°E, and 45.0° and 45.7°N. Vessel name and MMSI code, position (Latitude and Longitude), vessel speed and course were obtained from AIS message type 1. The ship characteristics such as the length, beam and draft were obtained from AIS message type 5.

A one-year record of AIS traffic data contains about 10^8^ records of moving ships. As acceleration, deceleration and changes in the sailing direction for large vessels are relatively slow processes and the depth of navigation channels also changes slowly and smoothly, the AIS information adequately describes the sailing line and (variations in) the speed of those vessels that produce large hydrodynamic loads to the seabed.

The assimilation of AIS and bathymetric data of the area in a GIS environment enabled a spatial analysis of parameters related to the formation of ship wakes. The basic quantities are the ship’s speed ($v$), width ($B$) and draft ($d$), and the width ($b$) and depth ($d$) of the channel. The blocking coefficient ($C$) is defined as the ratio of the approximate cross-sectional area of the ship ($bd$) to the cross-section of the channel $bd$. Another classic parameter is the depth-based Froude number $Fr=v/\sqrt{gh}$, where *g* is gravity acceleration and $H$ is the water depth. This parameter to some extent characterises the ship’s resistance to motion in shallow water^46^ and the appearance of its wake. The critical areas of ship traffic according to $C$ and $Fr$ were identified using representative values of these parameters. We additionally used a proxy ${V}_{i}=v/A$, where $A$ is the channel cross section area obtained from bathymetric data, that is useful for describing the hydrodynamic effects of a typical ship passing in the channel.

These quantities were calculated as follows. First, all entries that reflected non-moving ships and ships with a length less than 100 m were filtered out. As a result, a one-year record of AIS traffic data of moving ships longer than 100 m contains 2.2 × 10^6^ entries. Georeferenced point data transmitted by each ship that passed along the channel during the acquisition period were then interpolated with the IDW (Inverse Distance Weighted) method. The obtained raster was resampled along the centre line of the waterway with a resolution of 200 m. In other words, the ship’s velocity $v$ was interpolated for each 200 m long section of the channel. This spatial resolution roughly matches the original spatial resolution of the velocity data. For example, a ship sailing at 10 knots covers 200 m in 40 s.

Individual ship width and draft were extracted from the AIS traffic database. The dimensions of a representative ship (from the traffic statistics) and the bathymetric profile of each 200 m long channel section (from the GIS database) were used to calculate the local blocking coefficient $C$. The representative values of $Fr$ for each such section were obtained using the interpolated ship speed $v$ and local depth $d$. The quantity ${V}_{i}$ was calculated using channel cross section area obtained from bathymetric data.

As the main objects of study (solitary waves of depression) occur as single entities and involve only downward displacement of water surface, the classic concept of wave height as the distance from the maximum elevation at the crest to the maximum depression at the trough is not applicable. For such entities the wave height and amplitude coincide. To describe their height (amplitude), we employ the classic notion of solitary wave height ($H$) defined as the distance of the still water surface to the trough of the depression wave in the channel. For other undulations produced by the ship (including waves in the shallow lagoon) we rely on the classic concept of wave height as the crest to trough difference.

As vessel wakes propagate in short packets of waves that often have almost equal heights^21^, the concept of significant wave height is not applicable for their description; however, the notion of mean wave height is still meaningful. The described difference in the nature of ship wakes and wind wave fields suggest that a comparison of their impact should be done using certain integrated characteristics such as the total energy flux, wave-driven transport, or resuspension parameters. Pressure sensors were deployed to help quantify the impact of ship-driven perturbations remote from the channel. A shortcoming of this approach is that the reconstruction of wave properties from pressure data relies on linear wave theory, but the highest single waves of vessel wakes are substantially nonlinear^47^. For this class of motions, the attenuation of the pressure signal with water depth is negligible. The pressure signal reasonably follows the longer wake components in shallow water and is appropriate to capture the long-period perturbations associated with drawdown^25–28^.

**Figure 2:**
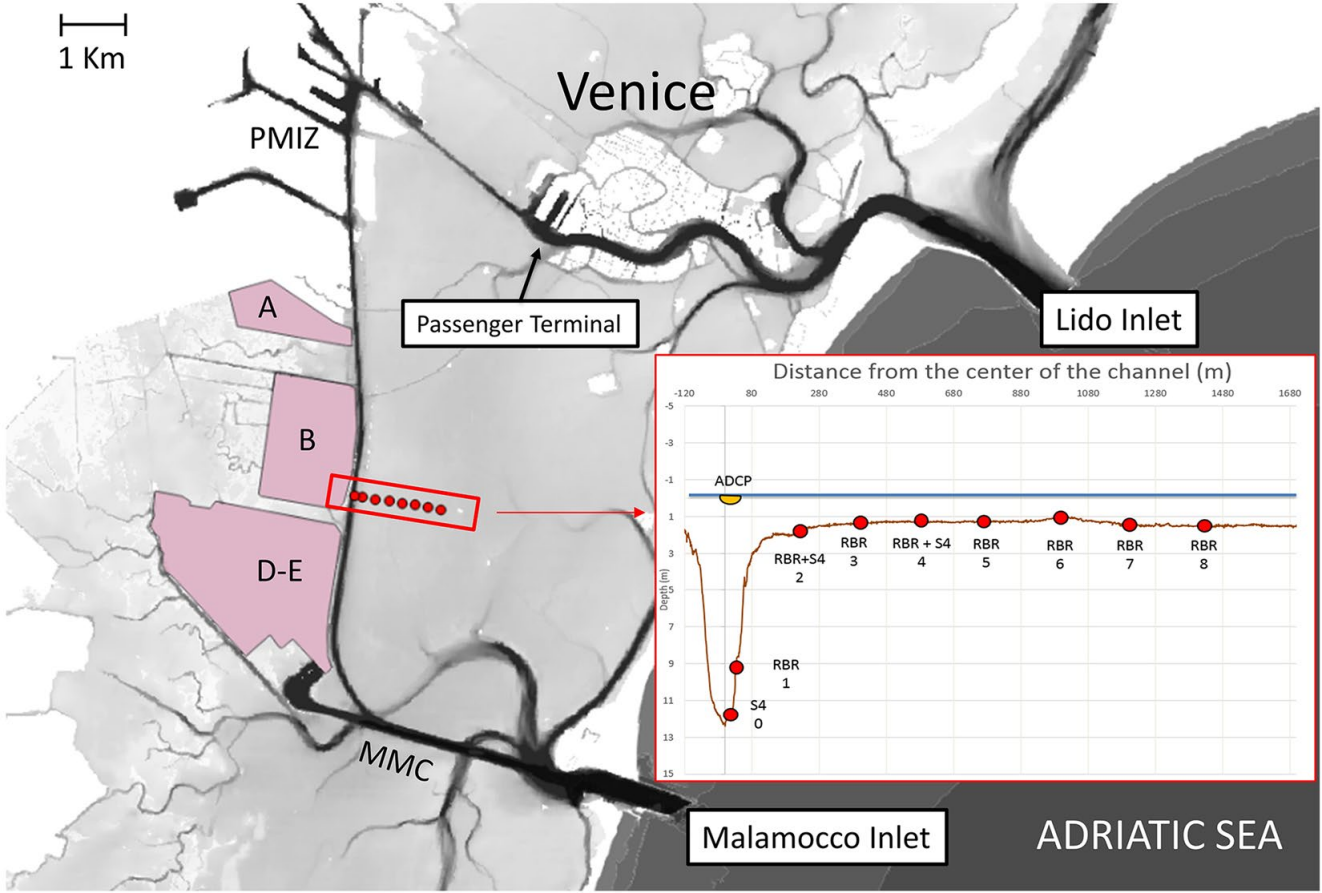
The study area with elevation data and bathymetry (in white, elevations of 1 m above local datum: Venezia Punta Salute that is −23.56 cm below the Italian official datum). The inset shows instrument positions and bathymetry of the study transect. A, B, D, and E are the reclaimed areas described in the text. The background map was created using free and open source geographic information system Quantum GIS software version 3.4.1. Open Source Geospatial Foundation Project. https://www.qgis.org. Original data, provided by Magistrato alle Acque di Venezia, were elaborated upon by Sarretta *et al*., as described in ref. ^39^. 10.1016/j.csr.2009.07.002. An open access version of the article can be downloaded from: 10.6084/m9.figshare.810481^71^. This article is licensed under a Creative Commons Attribution 4.0 International License.

Eight pressure sensors (stations 1–8 in Fig. 2) were deployed on the sub-tidal flat about 250 m apart along a line perpendicular to the navigation channel, starting from the eastern side of the fairway. The instruments used were RBR solo pressure sensors at 16 Hz sampling frequency. At four of the measurement stations on the sub-tidal flat (stations 2, 4, 6, and 8 in Fig. 2) turbidity and suspended particle concentration (SPM) were monitored continuously with Aqualogger 210TY turbidimeters (Aquatech, UK) at 0.2 Hz frequency. At stations 2 and 4 (Fig. 2), self-recording electromagnetic current meters (S4, InterOcean, USA) were deployed at 20 cm above bed level and set to sample with a frequency of 2 Hz. Another S4 device was deployed on the seabed on the eastern side of the channel (station 0 in Fig. 2) to measure water velocity and the magnitude of the depression wake in the near field during the passage of cruise ships in the MMC channel on 18–19 July 2015. A boat mounted acoustic Doppler current profiler (Teledyne-RDI 600 KHZ Workhorse Rio Grande ADCP) was also used at the surface above the S4 at station 0 to measure the profile of current speeds at the channel edge during the experiment period.

All events recorded by pressure sensors, turbidity loggers, and current meters deployed in the navigation channel and on the adjacent mudflats, were referenced to ships passing the measurement transect using the AIS information. Real time ship position data were also used to activate an automatic water sampling device remotely. The device was mounted on a floating platform close to the station 3 and set to collect 600 mL of water at 50 cm below the surface. This made it possible to obtain water samples during resuspension peaks created by ship wakes at times when we were not on site, and to collect samples for calibration of turbidity. The samples were used to calibrate turbidity sensors for the concentration of SPM determined as described in ref. ^28^. The grain size of SPM was determined by laser scattering using a LISST 100X particle size analyzer (Sequoia Scientific, USA) for field measurements and a Mastersizer 3000 granulometer (Malvern, UK), for laboratory determinations. The grain size ranges for the two instruments are 2–500 µm and 0.03–1000 µm respectively.

The variations in the coastline morphology were determined using GIS techniques. High-resolution information on rapid changes was acquired using drone surveys. Changes over longer time intervals were extracted from aerial and satellite images available from WMS services (Web Map Services) of the National and Regional cartographic portals. The relevant methodology is described in ref. ^43^.

## Results

### Unusual ship wakes in the venice lagoon

The motion of a ship in finite depths generates a depression region of the water surface called drawdown or Bernoulli wake^20,48^. In relatively shallow depths, this perturbation becomes evident as a region of lowered water level of nearly uniform depth^49,50^. The presence of this phenomenon causes the drawdown effect^49^. This effect is also called squat^51,52^ in inland waterways and navigation channels and is a well-known feature of sailing at depth Froude numbers larger than about 0.6 in shallow waters. This perturbation usually extends to a distance from the sailing line^53^ and may easily penetrate bays and harbors located adjacent to the sailing line and can cause unacceptable water level falls^54^ or damage^55^.

Under specific conditions, the depression signal may spread far from the waterway^28^. This happens especially if the channel is connected to shallow-water areas (e.g. tidal flats or water bodies similar to the Venice Lagoon) where the Bernoulli wake may excite strongly nonlinear long-living solitary waves of depression^25^. These waves penetrate to a distance of more than 1 km onto the sub-tidal flat^26^. They may undergo various transformations as they propagate, including shoaling-type effects^27^ and formation of bore-like phenomena^27^. These processes may influence the magnitude of the phenomena and amplify the depression generated, and therefore increase the intensity of erosion of both channel margins^43^ and areas in the shallows.

Out of more than 3000 commercial vessels that entered the PIMZ port through the MMC in 2016, according to the official statistics^8^, we monitored a sub-sample of all ship traffic over a period of 45 days. Wakes were recorded along a 1.5 km long transect over the sub-tidal flat on the east side of the MMC from April–May 2016. This time interval contains a total of 615 passages of large commercial vessels with a length of more than 100 m. At the channel margin, depression wakes had an average height of 0.52 m. The deepest depression (2.45 m) was excited by cargo vessel Xin Xia Men (length 280 m, width 40 m) on 26 April 2016. As squat of this magnitude seems unreasonable, it is likely that the classic Bernoulli wake in the vicinity of the ship hull (usually a drawdown of just a few tens cm) is amplified by shoaling and/or bathymetry-driven focusing on the channel margin.

This depression is, in essence, a part of the near-wave field of ship’s motion (a strongly nonlinear forced wave) and thus generally cannot be described in terms of travelling waves. Its original propagation direction cannot be quantified from the dataset at our disposal. As a consequence, we were not able to determine its approach angle to the channel margin and thus the exact trajectory, shoaling and refraction properties. Our dataset reflects the properties of the resulting wave-like disturbance on the channel margin. The resulting disturbance (that possibly drives a shallow-water Riemann wave^25^) propagates over the sub-tidal flat. As its propagation direction apparently does not match the orientation of the measurement line, the sensors, strictly speaking, detect the properties of different parts of the wave front. However, considering that the wave field is largely homogeneous along the navigation channel, it is safe to assume that our recordings adequately describe the changes to this depression wave over a long distance.

**Figure 3:**
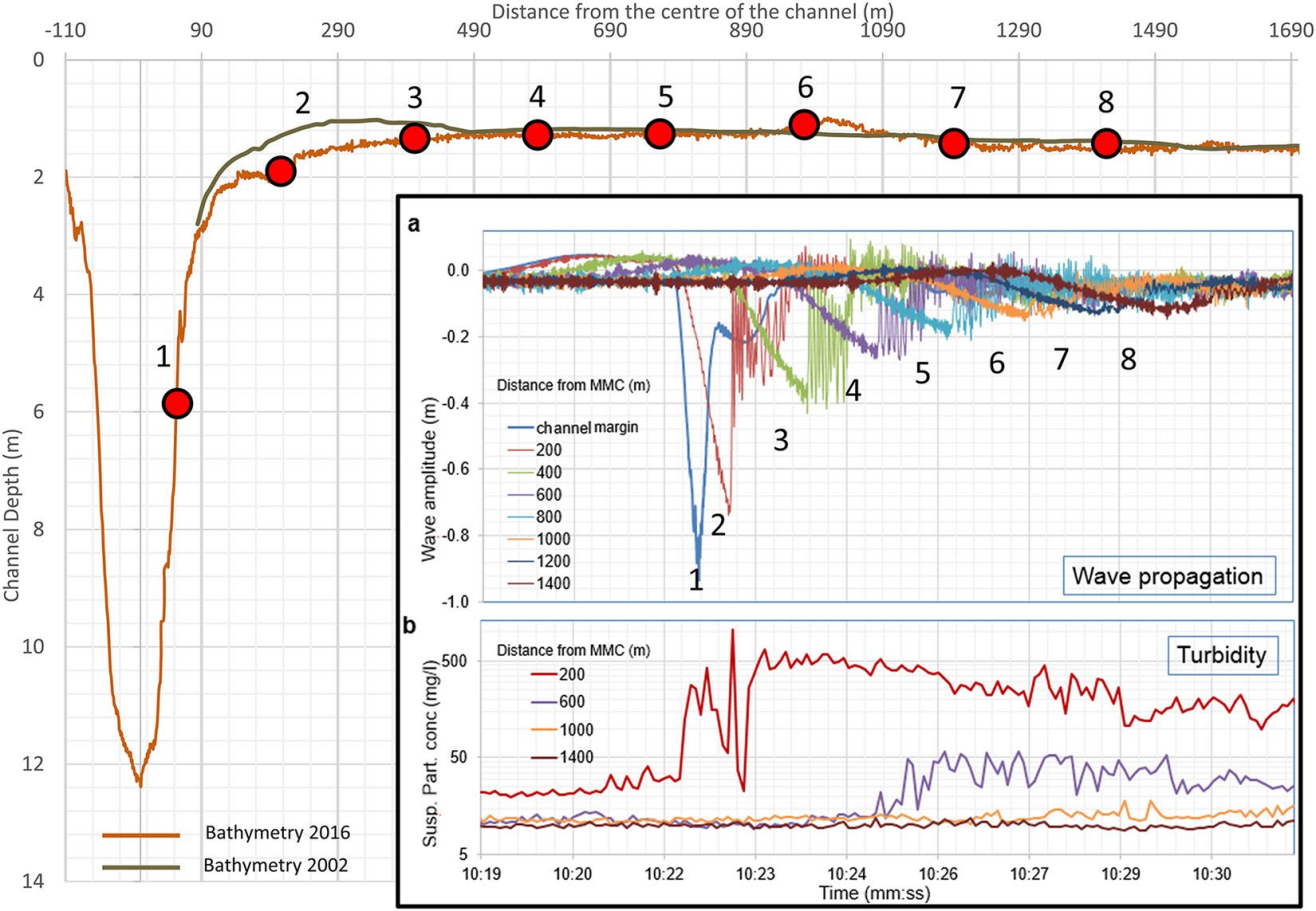
Instrument position along the investigated transect. Bathymetries acquired respectively in 2002 and 2016 are shown to emphasize the extent of erosion. (**a**) Propagation of the depression wave (as recorded by pressure sensors) generated by the passage of the ship Hellenic Spirit. (**b**) Time series of SPM concentration recorded at different distances from the channel after the passage of Hellenic Spirit. Both insets use the same colour code for single stations.

Even though the disturbance that travels into the lagoon may be a highly persistent entity^27^, its height gradually decreases as its energy is dissipated by the interaction with the seabed (Fig. 3a). The height of the depression wave reduces considerably as it propagates from the channel margin to the sub-tidal flat. Beyond a distance of about 800 m, the water surface dropdown in the depression maintains a more or less constant value of about 0.15 m (Fig. 3a). This amplitude is almost independent of the magnitude of the initial perturbation, as is also suggested by simulations^27^, and confirmed by other observations. The strong initial attenuation marks an intense dissipation of the wake energy within a certain range around the navigation channel.

Therefore, the most intense resuspension of bottom sediments occurs along the margin of the waterway and in the sub-tidal flat area near the channel. The SPM concentration in the water column at 200 m from the channel reaches 1000 mg/l after the perturbation. The intensity of resuspension is still considerable at a distance of 400 m from the channel where peaks of SPM concentration up to 58 mg/l were measured. This is much higher than typical natural concentrations (Fig. 3b). Resuspension remains moderate and the SPM concentration is not significantly altered further from the channel. Some of the sediment mobilized by each wake resettles soon after the event.

### The pattern of sediment transport

The fate of the sediment fraction that remains in suspension depends on the lagoon-scale motions induced jointly by successive ship transits and tidal currents as described in ref. ^28,29^. In particular, wakes of ships that enter the port in quick succession (within minutes of each other) often prevent particles settling and promote the efficient removal of sediment in suspension by the currents. This additional motion of the following ship (that is apparently associated with the alongshore transport of water by a set of deep depressions) can occur when the SPM concentration is still high from previous ship passages and causes a stepwise movement of sediment from the tidal flat towards the channel and thus exacerbates the silting of the channel.

**Figure 4:**
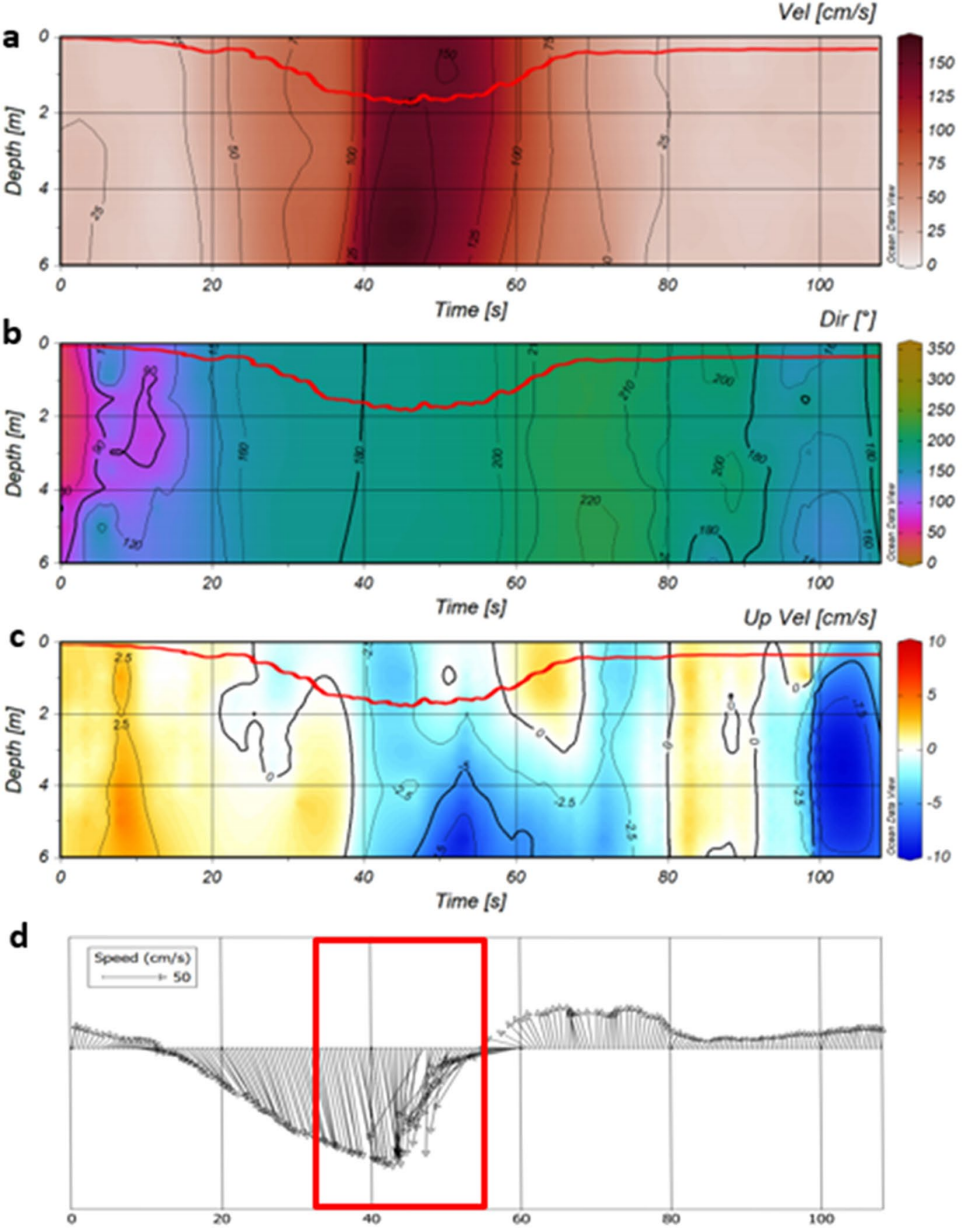
(**a**) Horizontal component of current velocity at the margin of the channel during the passage of MSC Musica (**b**) water movement direction, (**c**) vertical velocity (positive values indicate upward movements), (**d**) water velocity vector at the eastern margin of the channel bed during the passage of the MSC Musica. Vector lengths are proportional to the velocity magnitude. The red line in panels a, b and c indicates the water surface associated with the depression wave.

The complicated pattern of perturbations attributed to the water velocity associated with the passage of a ship in the channel can be to some extent interpreted by *in situ* measurements in the proximity of the sailing line. Data from one of these events, measured on the channel slope using both the ADCP (Fig. 4, panels a–c) and electromagnetic current meters S4 (Fig. 4d), shows the effects on the eastern bed margin during the passage of a cruise ship (MSC Musica, length 294 m, width 32 m) moving northward. The ADCP data set is limited to the first 6 m of the water column because of the intrinsic limitations related to side-lobe effects at the bottom. To complete the information, the near-bed (20 cm above the bottom) velocity from the S4 current meter is plotted in panel D.

The typical values of tidal currents in the area are 0.15 m/s. When a wake approaches, the horizontal speed increases abruptly up to 1.50 m/s (Fig. 4a, at 40 s). The direction of the flow is opposite to the direction of the vessel (Dir ≈ 180°, Fig. 4b). The passage of the drawdown (Bernoulli wake) forces the water to move from the sub-tidal flat towards the channel (Fig. 4b). This is evident after the velocity peak (about 60 s), when the current direction 225° indicates the movement of water from the sub-tidal flat to the channel. The bottom layer also initially moves towards the ship (Fig. 4d at 20–40 s) but later, the motion is directed perpendicularly to the channel (45–60 s, red framed subset). Both phases of the perturbation (reverse flow in the channel and motion towards the channel) affect the whole depth range covered by the ADCP.

The vertical component of current velocity shows an initial upward movement (Fig. 4c, at 8–10 s) with the largest values in the bottom layers. This feature probably reflects the arrival of a soliton-like elevation (so-called precursor soliton^23^) ahead of the ship. A downward movement, that can be naturally associated with the depression around the ship, dominates at 50–60 s. It is accompanied by flow from the sub-tidal flat to compensate for a local pressure drop in the channel. This flow has a component parallel to the channel as evidenced in the S4 data (Fig. 4d).

Immediately after the depression wave has moved further, the current velocity returns to values close to background levels. The described dynamics suggest that sediment resuspended from the channel margin and from the sub-tidal flat in the vicinity of the channel will generally be transported towards the navigation channel. Even though the transport time during each passage is fairly short (~20 s) and the transport range is about ten metres, this stepwise process is repeated thousands of times each year. Also, it may be enhanced by the cumulative effect resulting from consecutive ship transits^29^.

### Morphological impacts

As the typical length of the depression along its propagation direction is >100 m, this disturbance (if interpreted as a wave) is already a long wave in the navigation channel and is even more so when it travels over the tidal flat. It is therefore likely that the pressure sensors reliably reproduce the basic features of its shape even if some short-wave effects (occasionally occurring at its front^25^ and apparently reflecting the Kelvin wave system) may remain partly undetected.

The shape of the depression generated by the steadily sailing ship in such a channel, theoretically, should be a gradually lengthening and almost symmetric “valley” with relatively steep front and end that are separated by more or less constant-level trough^49,50^. This theoretical shape is specific to a weakly nonlinear framework^48,49^ and is apparently not created within the MMC as our data show that depression wakes on the channel margin are slightly asymmetric V-like features^26^. The further evolution of such waves is dictated by the properties of nonlinear shallow-water equations^56^. The depression wave is gradually modified due to the shoaling effect on the channel margins and over the shoals. It progressively loses its initial symmetry. The wave eventually transforms into a sawtooth-shape travelling Riemann wave^57^ with a very steep rear face (Fig. 3). The propagation of this steep wave front in natural conditions is usually accompanied with intensive energy dissipation, large horizontal water velocities and thus strong entrainment of the bottom sediment. The occurrence of many such events, essentially each large vessel, is reflected by morphological changes that affect the channel margins and the adjacent tidal and subtidal flats, marshlands and the artificial deposits of the reclaimed area^43^.

The joint impact of the described processes substantially depends on the nature of the seabed, the availability of finer sediment and the extent of the shallow-water areas adjacent to the channel. The described motions of water and associated transport of sediment lead to gradual erosion of the channel margins and tidal flats over the range where the depression waves cause considerable resuspension (as shown in the comparison of 2002 and 2016 bathymetries in Fig. 3). As described in ref. ^29^, the entrained materials are partly transported away from the site by background tidal currents but are also largely transported towards the channel (Supplementary Fig. S2) that is subjected to progressive filling with variable intensities depending on the erodibility of the channel edges and the tidal flat sediments. Results of this process are visible on high-resolution bathymetric surveys performed with multibeam echosounders that show the presence of erosional features on the channel slope and dredging marks on the channel bottom where sediment accumulates^58^.

**Figure 5:**
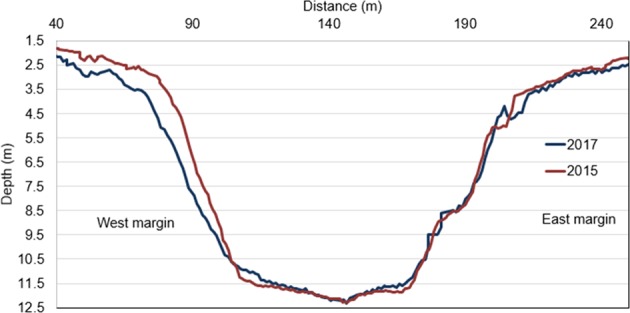
MMC Channel profiles from surveys of 2015 and 2017 showing considerable erosion on the western side. The western margin slope changed from 29° to 23°.

An example of the changes that occurred in the period 2015–2017 to the cross section of the MMC reported in Fig. 2 and Fig. 3 confirms the presence of the described pattern of changes. The western bank of the channel is narrow and consists of soft and easily erodible material. It is thus expected that under the hydrodynamic conditions caused by the ship wakes, this channel margin is able to be affected by considerable erosion over such a short time (Fig. 5).

**Figure 6:**
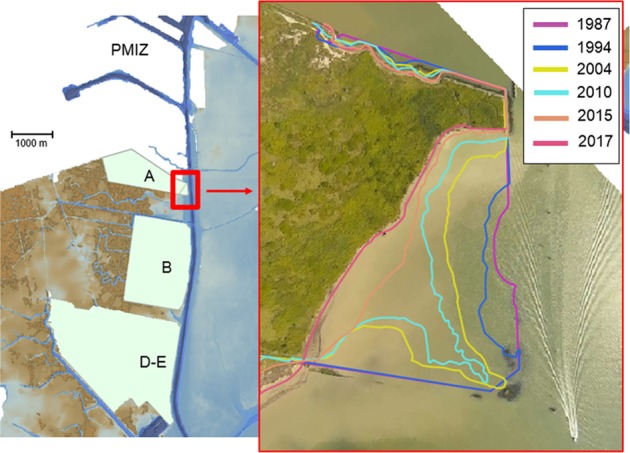
Coastline evolution of reclaimed area A on the western margin of the MMC in the period 1987–2017. The coloured curves outline the erosion of the shoreline from satellite and aerial images. Traces of a dismantled rip-rap protection are visible as dark patches at the bottom of the right-hand panel. The image of the reclaimed area A was extracted from aerial photos taken by the authors with an Unmanned Aerial Vehicle. The image with the coastline evolution of reclaimed area A was created using QuantumGIS software version 3.4.1. Open Source Geospatial Foundation Project. https://www.qgis.org.

The reclaimed land (areas A, B and D-E) to the west of the channel has been experiencing progressive shoreline retreat and over a period of 30 years underwent a rapid change which has no comparison in the recent morphological history of the lagoon^43^. The largest transformation affected the area A (Fig. 6) that was reclaimed in 1963 with materials dredged from the MMC. Similarly, to the other reclaimed areas its shoreline was initially protected by a rip-rap revetment. In accord with the processes described above, the erosion is not uniform in space and time. The total shoreline retreat in 54 years varies from about 70 to 220 m. The relevant regression rate (from 2 to 7 m/yr) significantly exceeds rates determined for reclaimed area B^43^.

**Figure 7:**
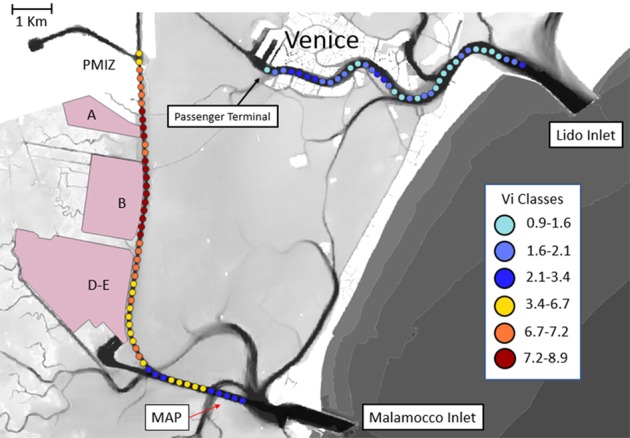
Longitudinal distribution of the quantity ${{\rm{V}}}_{{\rm{i}}}$ (coloured dots) along the MMC and Lido channels. The position of the turbidity gauge MAP is indicated. The background map was created using Quantum GIS software version 3.4.1. Open Source Geospatial Foundation Project. https://www.qgis.org starting from original data, provided by Magistrato alle Acque di Venezia, elaborated upon by Sarretta *et al*., as described in ref. ^39^. 10.1016/j.csr.2009.07.002. An open access version of the article can be downloaded from: 10.6084/m9.figshare.810481. This article is licensed under a Creative Commons Attribution 4.0 International License.

The relative roles of natural and ship-driven hydrodynamic activity on sediment transport and associated erosion and accumulation in the study area can be, to some extent, estimated based on the time during which the processes of different origin maintain elevated SPM concentrations. Time series of SPM concentration from a monitoring station positioned in the area of the lagoon are available on the website of a local institution (station MAP in Fig. 7)^59^ that is practically not affected by ship-induced depression waves. The SPM data set for this station covers a period of 10 years with a sampling frequency of 15 min. The recordings are validated for 90% of the total acquisition time. On an annual basis, the 95^th^ and 99^th^ percentiles of suspended particle concentration vary between 17.7 and 38.2 mg/l, and 30.6 and 110.1 mg/l, respectively (Supplementary Table S1). The same percentile values calculated from all recordings over the whole 10-year period are 22.2 and 50.5 mg/l, respectively^59^. These values are, by definition, representative of periods of high SPM concentration, that include summer algal blooms, and resuspension due to moderate wind events up to 10 m/s and major storm events. Therefore, only major resuspension events created by natural forcing, limited to 1% of the time, can approach or exceed the values typically found in the area impacted by the ship depression wakes at a distance of up to 0.5 km from the navigation channel. Ship wake induced high SPM concentrations are characterized by a long persistence (at least one hour) and occur on average 10 times a day. Therefore, extreme SPM concentrations are found in the vicinity of the navigation channel as long as there is ship traffic. Furthermore, in the area closer to the channel margin (up to a distance of 200 m), the SPM concentrations induced by ship wakes exceed the extreme natural values (expressed in terms of the 99 percentiles of naturally driven SPM concentrations) by at least one order of magnitude. It is therefore safe to conclude that the contribution of the natural forcing in the lagoon to the total erosion observed in the study site is of minor importance compared to the effects of ship traffic^28^.

### Channel sections with the largest impact

The existing research^25^ suggests that none of the classic parameters (blocking coefficient and the depth-based Froude number) nor their combination are able to properly describe the sailing regimes under which extremely strong depression waves are produced. The deepest depressions were generated by medium-size ships that sailed at fairly moderate Froude numbers^25^. A possible reason for this shortcoming is that none of these indicators combine the mutual relationships between the channel geometry and vessel speed. In other words, the separate use of these parameters without considering interactions does not enable the identification of the critical sectors along the waterways.

For this reason, we use the quantity ${V}_{i}=v/A$ [m^−1^s^−1^] to identify areas where larger hydrodynamic effects can be expected. Even though it is a dimensional measure, it combines the ship’s speed and the basic site-specific feature of the channel (its cross-section area) in a simple manner, and these two are known to affect the formation of strong wakes. From the definition of ${V}_{i}$ it follows that its larger values correspond to higher speeds and smaller channel cross-sections and this quantity thus could be a reasonable (at least qualitative) proxy of the chances for the formation of large wakes in different sections of the channels.

The values of ${V}_{i}$ (Fig. 7) along the two main waterways in the Venice Lagoon, MMC and Lido channel, were calculated, as described above, for each 200 m long section, for all ships longer than 100 m that entered the lagoon in 2016. Data for the Lido channel, which is still used as a main route for cruise ships, are presented for comparison. For the two waterways the MMC has the largest values of *V*_*i*_ in the middle section of this channel next to the reclamation areas A and B. Another short segment of large values of *V*_*i*_ is found at the turn of the channel in the south-eastern section of the waterway. The values of ${V}_{i}$ are much smaller in the channel that leads from the Lido inlet to the historic center of Venice. This difference can be explained by a combination of strict speed restrictions for cruise ships (AIS velocities are normally below 6 knots) that enter the city center and the relatively large cross-section of this waterway.

The patterns of spatial variation in the quantity ${V}_{i}$ explain, to a first approximation, the significant impacts of ship traffic found in the central part of the Venice Lagoon^43^. The largest values of ${V}_{i}$ are located in the MMC to the north of the study transect (Fig. 7). The east side of this part of the channel is protected by artificial shoal embankments. These structures were apparently designed to prevent ship wake propagation into the sub-tidal flat. High values of ${V}_{i}$ also occur along reclaimed area B where previous studies^43^ revealed rapid shoreline erosion. Our study site for ship wake measurements was specifically located in a segment with large values of ${V}_{i}$ and the frequent occurrence of large ship wakes^25^.

### The link between wave height and sailing regime

**Figure 8:**
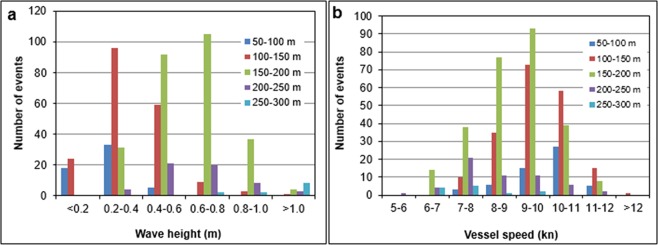
(**a**) Frequency distribution of depression wave height for vessels of different length, (**b**) Frequency distribution of vessel speed for vessels of different length.

It is natural to assume that the formation of depression wakes is mostly related to the vessel’s size, speed and hull shape. The relevant relationship is neither simple nor straightforward^25^. Figure 8 shows the frequency of depression wake amplitudes for different vessel dimensions. The largest disturbance in terms of magnitude and frequency is caused by vessels with a length in the range 150–200 m. This is the most frequent type of vessels in the MMC, accounting for 46% of the total number of identified ships. Ships from this category also pass the measurement cross-section with the highest speed (Fig. 8b). Specifically, 63% of such ships sail at 8–10 knots. It is thus likely that these vessels have the largest influence on the channel, sub-tidal flats and adjacent wetlands. However, as the distribution of the size and type of vessels is expected to vary in the future (e.g., if larger cruise ships use the MMC instead of the Lido inlet), this situation may change.

In an attempt to identify practically usable criteria for the magnitude of potential impacts of vessel wakes in the study area, we analysed the relationships between the amplitude of depression waves and the typically considered physical parameters of ships. The height of the depression wake is normally related to the depth Froude number $Fr\,$and blocking coefficient $C$. For example, Schoellhamer^12^ suggested that the quantity $F{r}^{2.4}{C}^{1.6}$ is the best predictor for the maximum height of ship waves. As this predictor has been derived for (almost linear) waves of elevation, it does not necessarily work for strongly nonlinear waves of depression in the Venice Lagoon.

**Figure 9:**
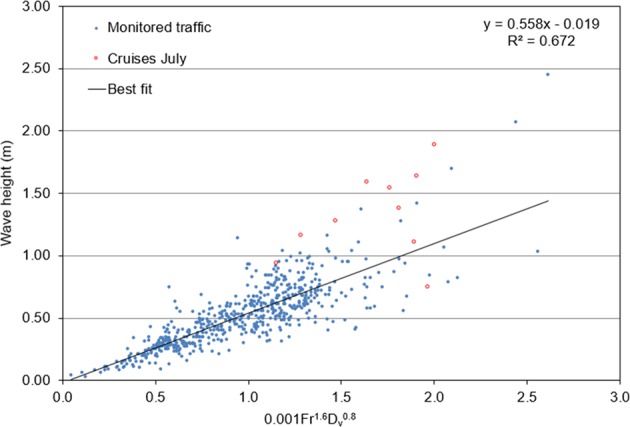
Scatterplot of depression wake heights (H) and the quantity $0.001\,{{Fr}}^{1.6}{{D}}_{{\rm{v}}}^{0.8}$. Red dots indicate cruise ship passages on 19 July 2015.

Following this line of thinking, we attempted to find a reasonable predictor for the height $H$ of depression waves as an empirical relationship that combines water displacement and the depth-based Froude number $Fr$. For each ship that passed the measurement section during the acquisition period we calculated the value of $Fr$ and $C$ as described in the Methods section, using the real time AIS data of velocity, draft, beam and length of the ships. We tested different combinations of powers of $Fr$ and $C$ as well as other geometrical parameters. Interestingly, an even better fit (in terms of a higher correlation coefficient ${R}^{2}$) than the optimal combination of $F{r}^{p}{C}^{q}\,$was obtained when $C$ was replaced by the displaced volume ${D}_{V}$. The optimal fit with experimental measurements of the height of the depression wave ($H$) (Fig. 9) is

1 $$1000\,H\approx 0.558\,F{r}^{1.6}{D}_{v}^{0.8}-0.019$$

The scatter of the values on different sides of Eq. (1) is quite limited for wave heights lower than 1 m. The cloud of data points is much wider for larger wave events (that are normally generated by larger vessels). A part of the scatter may be attributable to different tide levels. The variations in sea level in the entire lagoon are only partially accounted for in calculations of $Fr$. Other obvious contributors to the scatter are the distance of the ship route from the channel axis and different shapes of hulls.

The red dots in Fig. 9 reflect cruise ships measured on one day (19 July 2015), after the monitoring period of cargo ships. The data points for these vessels (that have dimensions and velocities comparable with those of typical larger cargo vessels) are also relatively strongly scattered, probably for reasons similar to those discussed above However, Fig. 9 suggests that the impact of this type of vessel (with lengths close to 300 m) falls within the largest range of magnitudes, as most wakes are >1 m. Therefore, for a confined channel like the MMC, the vessel length may play a specific role in the formation of large wakes. For a given blocking coefficient, a longer ship would excite a longer perturbation around the ship that in turn may drive a deeper depression on the channel margin.

## Discussion

It is well known that the high wake energy induced when a big ship navigates in coastal waters^60^, of limited depth^61^ or in a restricted channel^36^ is one of the main causes of erosion of side banks of channels^18^, nearby beaches^62^, microtidal estuaries^12^ and tidal flats^37^. Many ports worldwide, are experiencing problems related to vessel wakes that often are the main cause of sediment transport and erosion. They have been occurring and reported over many decades^23^ but have recently become much more evident and disturbing. This has happened partially because of the continued increase in the intensity of ship traffic and vessel size^24^ and partially because of the advances that have increased our capacity to detect and observe the consequences.

As mentioned above, these problems occur worldwide, including Charleston Harbor in the USA with an increased likelihood of shoreline erosion resulting from vessel wakes^38^, the Stockholm archipelago^36^ where one of the concerns is to regenerate the fine-sediment habitats that have been lost due to the impact of ship wakes, and in Australia with issues related to the optimization of shipping channel capacities for ports^63^. It is also widely recognised that wakes of some vessels may travel over long distances without substantial loss of energy and strongly impact shore sections many kilometres from the fairway, examples being Tallinn, Estonia^61^ and the Marlborough Sounds, New Zealand^64^.

The Venice Lagoon is a classic example where ship traffic can substantially modify the status and evolution of the habitat^43^. Ship wakes have the potential to strongly modify the cross section of the navigation channel and the adjacent shallow water areas^43,60^. In the light of similar research in other parts of the world^37,60,61^ showing the influence of vessel wakes as a dominant factor in morphodynamics^18^ due their relatively high intensity with respect to the natural forcing, this investigation also allows us to make the same inference regarding the situation in the Venice Lagoon. However, retreat rates of protected shoreline sections under the influence of ship wakes as high as those found in Venice in this study have not been previously recorded. The shoreline retreat in certain areas along the MMC channel can locally up to two times exceed previously measured erosion rates. To date, the rate of erosion shows no decrease over time.

The ongoing rise in the number and size of vessels serving the Venice port almost certainly extends the erosion effects in the lagoon, and our findings associated with the specific nature of ship wakes reveal a connection between ship parameters, velocity and erosion.

The principally new feature of our understanding of processes in the Venice Lagoon as described in this paper, is the unusual driver, namely, strongly nonlinear long-living solitary depression waves that resemble Riemann waves^25^. This hydrodynamic driver results in unusually strong vertical velocities in some sections of the wake (particularly the steep rear face) similar to depression-wave tsunamis. It is highly likely that this feature, together with a specific pattern of water motion generated by moving ships, is responsible for the morphological changes in large areas of the central lagoon basin.

Despite the relative uncertainty of estimates of the depression wave heights for larger vessels, Fig. 9 indicates that feasible management options to address the impacts of ships with given dimensions may exist. Firstly, vessel speed can be managed in order to limit depth-based Froude numbers. Secondly, the hull geometry of ships could be improved in the future.

Management in terms of the Froude number requires highly accurate bathymetry data, information about water level during the ship passage across the lagoon (according to tides) and real-time positioning. While the first two sets of information can be fairly easily retrieved or predicted, the management of ship speed is more complicated. Existing regulations for vessel traffic in the MMC industrial channel^65^ only establish general criteria based on safety issues and do not involve any options to minimise the impacts of ship traffic. The regulations also do not prescribe any speed limit along most of the channel, only prescribing a speed limit at the entrance to the industrial port.

A rational regulatory approach that also enhances protection of the environment does not necessarily imply a contraction of port activities. Such regulation could lead to immediate positive effects due to: (i) less erosion and therefore savings in dredging costs; (ii) the ability to adopt ship-specific speed limits (that could allow certain low-impact vessels to move faster and foster future improvements in vessel design); (iii) an increase in the payload for certain types of ships. As a first approximation, express estimates based on Eq. (1) (or more refined versions) have the potential to drive changes in the regulations for ship traffic towards a more sustainable, safe and efficient vessel traffic management system. Such systems would also reduce both the cost and the impact of dredging^63^.

The results of our research suggest that progress in this direction has to become a priority if the current intention to redirect cruise ships into the MMC is implemented. Unless carefully and specifically managed, this scenario of combined cruise, commercial and industrial shipping will lead to increased negative impacts of ship traffic in parts of the Venice Lagoon

Figure 9 clearly indicates that even based on a simplified parameter, the hydrodynamics and morphological impacts of increased ship traffic in the MMC on critical areas of the Venice Lagoon are likely to be very significant. The largest changes are expected in the areas that are not yet in morphological equilibrium with the stressors, such as the tidal flat in the vicinity of the MMC and the shorelines of the reclaimed areas. Figure 9 also indicates that the projected transit of cruise ships in the MMC would considerably increase the impacts on the morphology of the channel and sub-tidal flats.

According to the statistics of the port of Venice^8^, if all cruise ships are redirected through the MMC, about 500 additional vessels (i.e., 1000 transits) will be added to existing traffic in the waterway each year. If no action is taken to limit depression wave height, most cruise ships are expected to produce very large wakes. This rerouting, therefore, may lead to a significant increase in the erosion of areas that are already shown to be susceptible to natural and anthropogenic processes such as sea level rise and subsidence^66^.

Strong impacts on the seabed may also have equally concerning side effects. The Venice Lagoon has been used as a port for many centuries and for a variety of other purposes. Additional intense erosion may adversely impact submerged historic and prehistoric cultural relics^30^. As a result of industrial activities in the surrounding area, lagoon sediments are known to accumulate concentrations of contaminants that could be released into the environment by erosion and remobilization of sediments. The almost continuous resuspension of sediment in the area can redistribute toxic pollutants of industrial origin from the lagoon sediments^67,68^ and reclaimed areas. Moreover the pumping effect of ship wakes on ground water in contaminated aquifers in the industrial area in contact with the channel bed^69^ can cause the release of harmful contaminants and eventual effects on the biota.

Finally, it is important to underline the existence of many other similar situations worldwide where navigation channels cut through lagoons, estuaries or extensive nearshore areas that are surrounded by wide shallow water bodies. While the threats associated with the direct impact of the classic Kelvin and Bernoulli wakes and the remote impact of nonlinear waves of elevation and various wave packets have been widely recognised for a long time^70^, the presence of strongly nonlinear solitary waves of depression in certain situations was only discovered a few years ago^25^. As wave physics is universal, it is likely that such waves are often present in many water bodies all over the world. This highlights the need to reconsider the estimates of potential environmental impacts of ship traffic in all similar situations.

## Supplementary information

Supplementary information

## Data Availability

The datasets generated and/or analysed during the current study are available from the corresponding author on reasonable request.
