# Supplementary material for: The effects of ship wakes in the Venice Lagoon and implications for the sustainability of shipping in coastal waters
Source: Sci Rep. 2019 Dec 12;9:19014. doi: 10.1038/s41598-019-55238-z (PMC6908581; doi:10.1038/s41598-019-55238-z)

Paper title:

The effects of ship wakes in the Venice Lagoon and implications for the sustainability of shipping in coastal waters

Gian Marco Scarpa[1,2,*], Luca Zaggia[2], Giorgia Manfè[2], Giuliano Lorenzetti[2], Kevin Parnell[3], Tarmo Soomere[3], John Rapaglia[4], and Emanuela Molinaroli[1]

Supplementary Information

Supplementary Table S1

Statistics of suspended particles matter concentration in the MAP measurement station (Fig.7). Data acquired in continuous during the period 2005-2015 with a sampling frequency of 15 mins.

	**Apr 2005-May 2006**	**Apr 2006-May 2007**	**Apr 2007-May 2008**	**Apr 2008-May 2009**	**Apr 2009-May 2010**	**Apr 2010-May 2011**	**Apr 2011-May 2012**	**Apr 2012-May 2013**	**Apr 2013-May 2014**	**Apr 2014-May 2015**	**Apr 2006-May 2015**
Ntot	24968	31057	33311	25920	33366	32609.0	31452	31576	33665	27115	308355
Mean	9.0	13.5	11.9	16.2	10.6	9	7.2	7.3	7.4	7.2	9.2
Std. Dev.	6.7	8.6	12.1	17.4	13.5	7.6	11.1	7.3	6.3	10.2	10.6
Max.	91.1	193.1	158.9	230.7	299.9	141.7	153.4	195.0	86.3	299.0	299.9
Min.	2.0	1.6	0.3	0.7	1.3	1.8	0.3	0.5	0.3	0.2	0.2
10th percentile	4.5	6.5	3.7	6.7	4.7	4.2	2.0	2.4	2.2	2.4	3.1
25th percentile	5.5	8.6	6.2	8.8	5.7	5.5	3.2	3.4	3.6	3.5	4.6
30th percentile	5.8	9.3	6.9	9.4		5.9	3.6	3.7	4.0	3.9	5.0
50th percentile	7.0	11.7	9.3	12.1	7.5	7.5	4.8	5.1	5.8	5.3	6.8
70th percentile	9.0	14.8	12.2	15.9		9.9	6.3	7.4	8.0	7.4	9.4
75th percentile	9.8	15.9	13.2	17.3	10.7	10.7	6.9	8.2	9.0	8.3	10.4
90th percentile	15.3	21.6	20.3	25.8	17.2	14.6	10.6	14.5	14.8	12.6	16.2
95th percentile	21.3	27.0	28.7	38.2	24.9	18.8	17.7	19.9	18.7	16.8	22.2
99th percentile	36.8	44.5	69.0	110.1	59.0	42.2	65.6	36.3	30.6	32.5	50.5

Supplementary Figure S2

Depression wave generated by the transit of a cruise ship in the Malamocco Marghera Channel. The arrows show the pattern of water and sediment transport during the passage of the perturbation. In the first part of the event, corresponding to the interval between the start of the perturbation and the passage of the trough, the fast current moves large amount of sediment towards the channel. The steep and turbulent rear edge of the wave coming immediately after, moves sediments in a direction almost parallel to the shore causing the erosion of the reclaimed area, seen on the left (image by the authors).

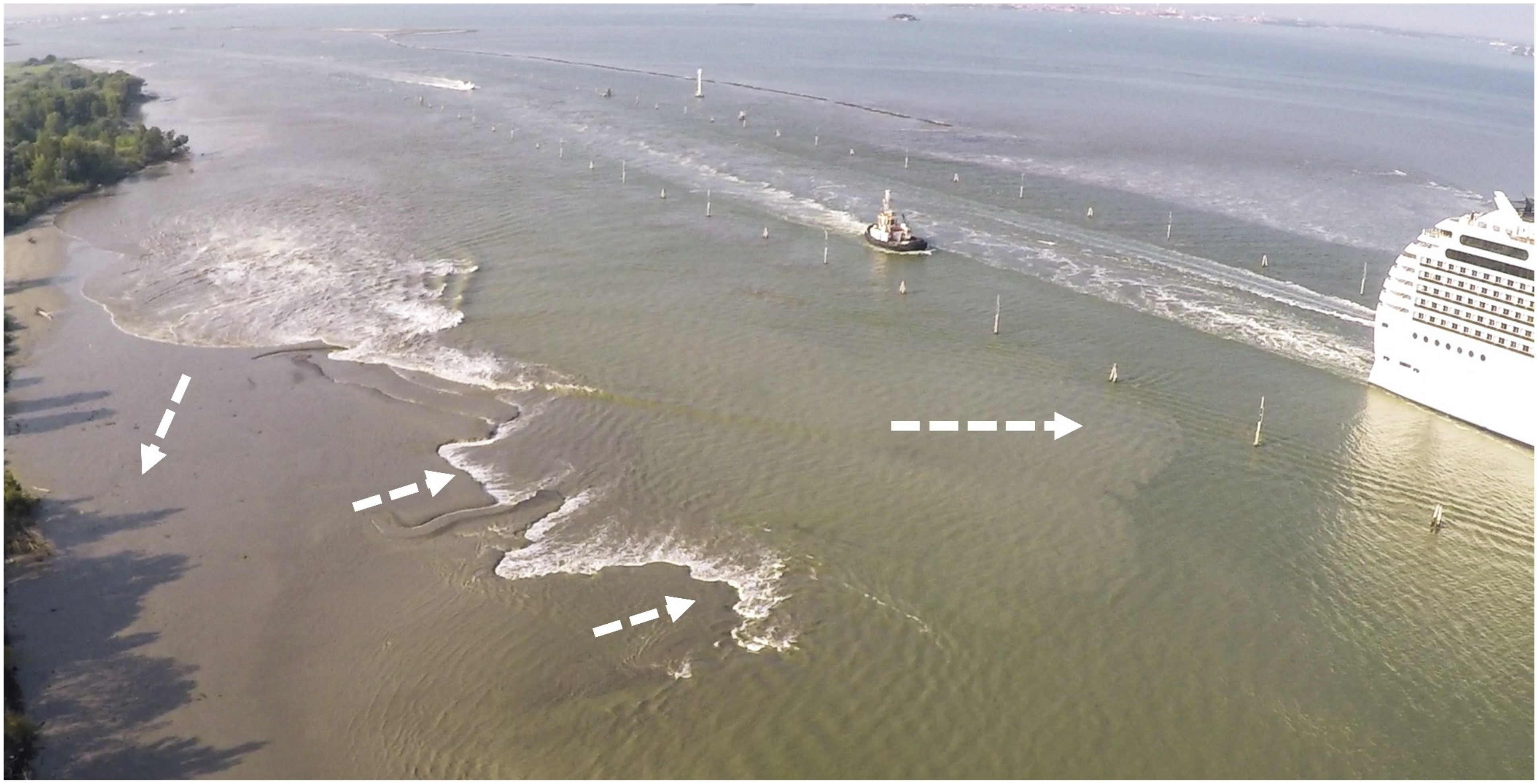

Supplement: Supplementary file 1 — Supplementary information [file 41598_2019_55238_MOESM1_ESM.pdf]
